# Closed Versus Open Surgical Exposure of Palatally Displaced Canines: A Systematic Review and Meta-Analysis

**DOI:** 10.7759/cureus.112792

**Published:** 2026-07-16

**Authors:** Yousef T Aldoseri, Dalal Alkhawari, Yousef Ashour, Mohammad F Behbehani, Ali F Khajah, Latifah A Alali, Farah Alsalem, Hanan T Alenezi, Saad Almutairi, Reem A Aldhafeeri, Mubarak Alshammari, Mohammad Shanat, Ammar A Almekhyal, Yousef Alajmi

**Affiliations:** 1 Orthodontics, Riyadh Elm University, Kuwait, KWT; 2 Dentistry, Ministry of Health, Kuwait, Kuwait City, KWT; 3 Dentistry, Ministry of Health, Kuwait, Al Ahmadi, KWT; 4 Dentistry, Ministry of Health, Kuwait, Aljahra, KWT; 5 Dentistry, Oyoun Polyclinic, Jahra, KWT

**Keywords:** closed eruption, impacted canine, meta-analysis, open eruption, orthodontic traction, palatally displaced canine, surgical exposure

## Abstract

Palatally displaced canines (PDCs) are a common orthodontic anomaly that frequently require surgical exposure to facilitate orthodontic alignment*. *Two primary techniques exist: open surgical exposure, where the canine is left to erupt spontaneously, and closed surgical exposure, where an attachment is bonded for immediate orthodontic traction. This meta-analysis aims to compare the efficacy of closed versus open surgical exposure for PDCs regarding pain, surgical time, complications, eruption time, and patient-reported outcomes. We systematically searched the following databases: PubMed, Cochrane Library, Scopus, and Web of Science. Studies were included if they compared open versus closed surgical exposure techniques for palatally displaced canines. R software was used for statistical analysis. Continuous outcomes were analyzed as mean differences (MD) or standardized mean differences (SMD) and dichotomous data as risk ratios (RR), with 95% confidence intervals (CI). Our review included six studies with 450 participants. Pooled analysis demonstrated that closed exposure was associated with significantly less pain at 1 day (SMD=-0.39, 95% CI -0.75 to -0.04) and less discomfort (SMD=-0.25, 95% CI -0.42 to -0.07). However, closed exposure required significantly longer surgical time (MD=5.09 minutes, 95% CI 1.35 to 8.82) and longer eruption time (MD=3.37 months, 95% CI 2.02 to 4.73). No significant differences were observed for analgesic consumption, gingival recession, or overall complications. Closed surgical exposure offers advantages in early postoperative pain and discomfort but requires longer surgical and eruption times. Both techniques appear comparable regarding complications and periodontal outcomes. The choice of technique should be individualized based on clinical factors, patient preference, and clinician expertise. Future large-scale RCTs with standardized protocols are warranted to further refine these findings.

## Introduction and background

Managing palatally displaced canines (PDCs) is often considered a significant clinical challenge in orthodontics. This anomaly is observed in roughly 1% to 3% of individuals, with a female-to-male predilection of 2:1 [1, 2]. Maxillary canines are essential for maintaining the continuity of the dental arch, supporting lateral excursions, and enhancing overall facial and smile aesthetics [3].

Early interceptive approaches, including primary canine extraction and arch expansion, are often employed to avert impaction and mitigate risks such as loss of arch length, dental asymmetry, and adjacent root resorption [4-6]. If these early interventions fail, or if a PDC is diagnosed after impaction has occurred, the conventional approach requires surgical exposure of the impacted crown, subsequently followed by orthodontic traction to align the tooth within the arch [7].

Two primary surgical methods are used to expose a palatally displaced canine: closed and open. The closed technique is a standardized procedure that involves full-flap repositioning, complete recovery of the impacted canine after exposure, and bonding an attachment to the crown. Orthodontic force is applied shortly after surgery, and the canine is moved beneath the palatal mucosa. In the open technique, a similar amount of bone covering the canine crown is removed; however, the crown is left exposed to the oral cavity, and the opening is maintained using a surgical dressing [2].

 The subsequent treatment approach depends on whether an attachment is bonded to the exposed tooth for immediate orthodontic traction or if the tooth is allowed to erupt spontaneously. In either case, the tooth erupts above the palatal mucosa. The open technique may or may not involve flap surgery [8, 9]. Various dressings are used in open surgical exposure, including Glass-Ionomer cement (GIC) and the so-called GOPEX technique [9, 10].

The choice between open and closed techniques remains controversial. Open surgical exposure avoids the need for immediate attachment placement, reduces surgical time, and allows for natural eruption [2]. Conversely, closed surgical exposure provides better control over the direction of eruption, reduces the risk of gingival overgrowth, and may lead to more predictable outcomes [2]. An earlier Cochrane review included only a limited number of studies (three studies) and lacked comprehensive meta-analyses for several outcomes important to patients [2]. Since that Cochrane review was published, new high-quality randomized controlled trials (RCTs) have emerged [11, 12], offering an opportunity to update the existing evidence. Accordingly, we performed this systematic review and meta-analysis to compare the efficacy and safety of closed versus open surgical exposure techniques for PDCs, focusing on pain, operative time, complications, and eruption duration.

## Review

Methods

Study Registration

The study protocol was registered on PROSPERO (CRD420261364910). This systematic review adhered to the Preferred Reporting Items for Systematic Reviews and Meta-Analyses (PRISMA) 2020 statement standards [13] and was conducted in accordance with the Cochrane Handbook for Systematic Reviews [14]. The PRISMA checklist is provided in the supplementary appendix.

Literature Search and Study Selection

We systematically searched the following databases: PubMed, Cochrane Library, Scopus, and Web of Science up to 19 March 2026, using these search terms in PubMed: ( canine* OR cuspid* OR "Eye tooth" ) AND ( impact* OR unerupt* OR ectopic OR displaced OR embedded) AND ( “surgical exposure*” OR "open exposure*" OR "open surgical exposure*" OR "open eruption" OR gingivectomy OR "apically positioned flap" OR "apically repositioned flap" OR "closed exposure*" OR "closed surgical exposure*" OR "closed eruption" OR "orthodontic traction" OR "forced eruption" OR "bonded attachment*" OR "gold chain*" OR uncover* OR "guided eruption" OR Fenestration OR "window technique" OR "Surgical technique" ) AND ( random* OR control* OR trial* OR "split mouth" OR "split-mouth" OR RCT ). Detailed search strategies are displayed in Table 1.

**Table 1:** Search strategies

Databases	Search strategies	Results	Limitations
PubMed	( canine* OR cuspid* OR "Eye tooth" ) AND ( impact* OR unerupt* OR ectopic OR displaced OR embedded) AND ( “surgical exposure*” OR "open exposure*" OR "open surgical exposure*" OR "open eruption" OR gingivectomy OR "apically positioned flap" OR "apically repositioned flap" OR "closed exposure*" OR "closed surgical exposure*" OR "closed eruption" OR "orthodontic traction" OR "forced eruption" OR "bonded attachment*" OR "gold chain*" OR uncover* OR "guided eruption" OR Fenestration OR "window technique" OR "Surgical technique" ) AND ( random* OR control* OR trial* OR "split mouth" OR "split-mouth" OR RCT ) (All fields)	136	No limitations applied
Web of science	( canine* OR cuspid* OR "Eye tooth" ) AND ( impact* OR unerupt* OR ectopic OR displaced OR embedded) AND ( “surgical exposure*” OR "open exposure*" OR "open surgical exposure*" OR "open eruption" OR gingivectomy OR "apically positioned flap" OR "apically repositioned flap" OR "closed exposure*" OR "closed surgical exposure*" OR "closed eruption" OR "orthodontic traction" OR "forced eruption" OR "bonded attachment*" OR "gold chain*" OR uncover* OR "guided eruption" OR Fenestration OR "window technique" OR "Surgical technique" ) AND ( random* OR control* OR trial* OR "split mouth" OR "split-mouth" OR RCT ) (All fields)	125	No limitations applied
Cochrane library	( canine* OR cuspid* OR "Eye tooth" ) AND ( impact* OR unerupt* OR ectopic OR displaced OR embedded) AND ( “surgical exposure*” OR "open exposure*" OR "open surgical exposure*" OR "open eruption" OR gingivectomy OR "apically positioned flap" OR "apically repositioned flap" OR "closed exposure*" OR "closed surgical exposure*" OR "closed eruption" OR "orthodontic traction" OR "forced eruption" OR "bonded attachment*" OR "gold chain*" OR uncover* OR "guided eruption" OR Fenestration OR "window technique" OR "Surgical technique" ) AND ( random* OR control* OR trial* OR "split mouth" OR "split-mouth" OR RCT ) (All text)	46	No limitations applied
Scopus	( canine* OR cuspid* OR "Eye tooth" ) AND ( impact* OR unerupt* OR ectopic OR displaced OR embedded) AND ( “surgical exposure*” OR "open exposure*" OR "open surgical exposure*" OR "open eruption" OR gingivectomy OR "apically positioned flap" OR "apically repositioned flap" OR "closed exposure*" OR "closed surgical exposure*" OR "closed eruption" OR "orthodontic traction" OR "forced eruption" OR "bonded attachment*" OR "gold chain*" OR uncover* OR "guided eruption" OR Fenestration OR "window technique" OR "Surgical technique" ) AND ( random* OR control* OR trial* OR "split mouth" OR "split-mouth" OR RCT ) (Article title, Abstract, Keywords)	208	No limitations applied

We used EndNote software (Clarivate Analytics, PA, USA) to remove duplicates [15]. References were uploaded to Rayyan, where two independent reviewers conducted title/abstract screening followed by full-text screening for final eligibility [16]. Any conflict was resolved by a third author’s decision.

Eligibility Criteria

We included studies that met the following population, intervention, comparison, outcomes, and study design (PICOS) criteria [17].

Randomized controlled trials and prospective controlled clinical studies that compared closed surgical exposure with open surgical exposure for palatally displaced maxillary canines in human participants undergoing combined surgical-orthodontic management were included. Studies were eligible when they enrolled children, adolescents, or adults with unilateral or bilateral PDCs and reported at least one prespecified outcome of interest, including postoperative pain, discomfort, analgesic consumption, surgery time, overall complications, periodontal outcomes, or eruption duration.

The intervention of interest was closed surgical exposure, defined as surgical exposure with attachment bonding followed by flap repositioning so that the impacted canine continued eruption beneath the palatal mucosa. The comparator was open surgical exposure, defined as surgical uncovering of the canine crown with the crown left exposed to the oral cavity, either to allow spontaneous eruption or to facilitate subsequent orthodontic traction. Variations in dressings or flap design were accepted provided that the overall surgical concept corresponded to one of these two approaches.

Narrative reviews, systematic reviews, case reports, case series, retrospective observational studies, editorials, conference abstracts without sufficient data for extraction, animal studies, and laboratory studies were excluded. We also excluded studies that did not distinguish between palatal and buccal canine displacement, studies in which the exposure technique could not be clearly categorized as open or closed, studies lacking a comparison group, and duplicate publications reporting the same outcome data without additional relevant information. When multiple reports described the same trial cohort, they were linked together and counted as a single study for participant totals.

No restriction was applied with respect to year of publication. Only studies available in English were included.

Data Extraction

Two independent authors conducted data extraction using a standardized Excel sheet. Extracted data included study characteristics (population, country, study design, sample size, follow-up duration, technique details), intervention details (flap type, exposure, attachment type, timing of traction), baseline characteristics (age, sex, canine sector, angulation, vertical position, previous orthodontic treatment), and outcome measurements as defined in the included studies.

Quality Assessment

Two independent authors assessed the risk of bias in included RCTs using the Cochrane Risk of Bias 2 (ROB2) tool, evaluating five domains: randomization process, deviations from intended interventions, missing outcome data, measurement of the outcome, and selection of reported results [18]. Conflicts were resolved by a third author. Results were visualized using the RobVis web tool [19].

Statistical Analysis

All statistical analyses were performed using R software version 4.5.0 (R Foundation for Statistical Computing, Vienna, Austria) with the meta and metasens packages [20]. For continuous outcomes, pooled effect estimates were calculated using the mean difference (MD) or the standardized mean difference (SMD), as appropriate. The MD was used when outcomes were measured on the same scale, while the SMD was applied when different scales were used across studies. For dichotomous outcomes, pooled effect estimates were expressed as risk ratios (RR). For RR analyses, pooled estimates were calculated using the Mantel-Haenszel method under a common-effect model and the inverse-variance method under a random-effects model. Between-study variance (τ²) was estimated using the restricted maximum-likelihood (REML) method, and confidence intervals for τ² and τ were derived using the Q-profile method. The I² statistic was calculated based on the Cochran Q statistic to quantify heterogeneity. For MD and SMD analyses, the inverse-variance method was used. For SMD, effect sizes were calculated as Hedges' g (bias-corrected standardized mean difference using exact formulae). A random-effects model was used as the primary analytical approach. Between-study heterogeneity was assessed using the Cochran Q test, I² statistic, and τ², with τ² estimated using the restricted maximum-likelihood (REML) method and confidence intervals derived using the Q-profile method. Heterogeneity was interpreted according to established thresholds (low≈25%, moderate≈50%, high≈75%).

Pre-specified subgroup analyses were conducted according to follow-up duration and laterality. Differences between subgroups were examined using a chi-square test for interaction. Sensitivity analysis was performed using a leave-one-out approach, sequentially omitting each study and recalculating the pooled effect estimate to assess robustness. In cases where heterogeneity remained substantial despite leave-one-out analysis, Baujat plots were constructed to identify studies contributing disproportionately to overall heterogeneity and effect size influence [21]. The Baujat plot is a diagnostic scatter plot in which each study is positioned according to its contribution to the overall Cochran's Q statistic (x-axis) and its influence on the pooled effect estimate (y-axis); studies appearing in the upper-right quadrant simultaneously drive heterogeneity and exert the greatest influence on the overall result [21].

Publication bias and small-study effects were assessed using the Doi plot and the Luis Furuya-Kanamori (LFK) index [22]. The Doi plot arranges studies by effect size and plots the corresponding normal quantile scores on the y-axis; a symmetrical isosceles triangle shape indicates the absence of publication bias, whereas asymmetry suggests potential selective reporting of results [22]. The LFK index quantifies this asymmetry numerically, with values within ±1 indicating no asymmetry, values between ±1 and ±2 indicating minor asymmetry, and values beyond ±2 indicating major asymmetry [22]. In cases of major asymmetry (LFK index beyond ±2), the trim-and-fill method was applied to estimate potentially missing studies and to generate adjusted pooled estimates [23]. This method iteratively removes smaller studies causing asymmetry (trim), estimates a corrected symmetric pooled effect, and then imputes hypothetical mirror-image studies on the opposite side of the distribution (fill), yielding a bias-adjusted pooled estimate [23].

Results

Literature Search

**Figure 1:**
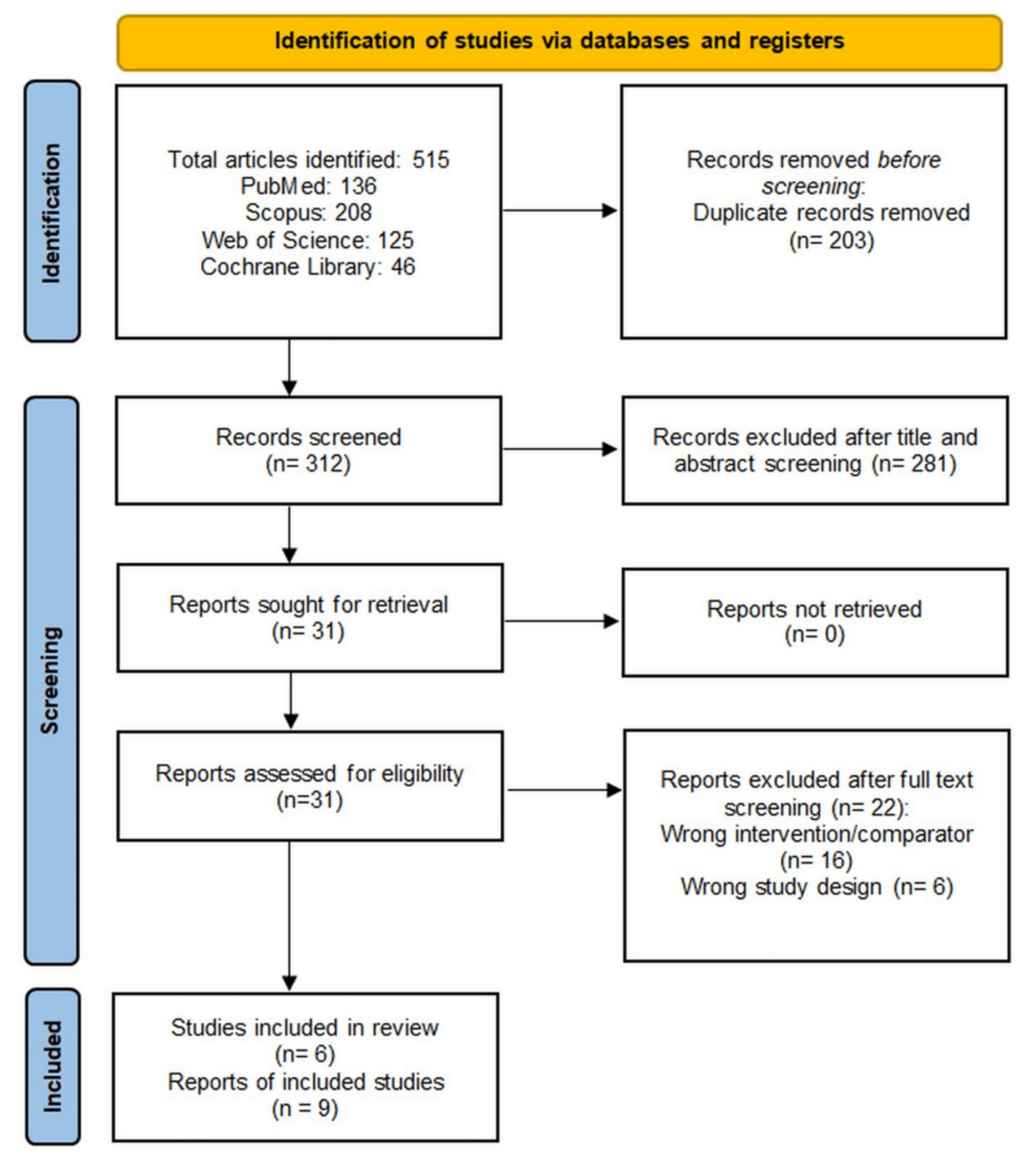
PRISMA flow chart for the systematic search and selection process Studies included in the review [10-12, 25, 26, 29]. Reports of included studies [10-12, 24-29].

Finally, six distinct clinical trials met the inclusion criteria, which were described across nine separate published reports [10-12, 24-29]. Specifically, the trials by Björksved et al. yielded two reports [10, 24], and the trials by Parkin et al. yielded three reports [26-28], while the remaining four trials yielded one report each [11, 12, 25, 29]. The PRISMA flow diagram is shown in Figure 1.

Study Characteristics

Six studies were included in this review with a total of 450 participants [10-12, 25, 26, 29]. Sample size ranged between 32 and 119 patients. All studies focused on maxillary palatally displaced canines in pediatric and adolescent populations. Surgical techniques varied across studies. For open surgical exposure (OSE), common approaches included a full-thickness mucoperiosteal flap with bone removal, a window of tissue overlying the tooth removed with a punch or scalpel, and allowing spontaneous eruption without immediate traction. For closed surgical exposure (CSE), approaches included raising a flap, bonding an attachment (GOPEX or GIC) to the canine, and initiating orthodontic traction within one to three weeks post-surgery. The mean age of the participants ranged from 12.9 to 17.6 years. Study and baseline characteristics are presented in Tables 1, 2, and the surgical and intervention details of each study are in Table 3, while the baseline demographic and clinical characteristics of the participants are detailed in Table 2. 

**Table 2:** Summary of the included studies It is important to note that while the PRISMA diagram indicates nine reports, several of these represent multiple publications originating from the same trial cohorts. Specifically, Björksved et al. [10, 24] published two separate reports from the same RCT cohort (2018 and 2021), and Parkin et al. [26-28] published three reports from the same RCT cohort (2012, 2013, and 2015). These linked reports were grouped by their respective parent trials and counted as a single study each for the purpose of participant totals, ensuring that patient cohorts were not double-counted in the meta-analysis. All six distinct trials were included in the meta-analysis where data were available for each outcome. DS2M1–DS3M2: dental development stages according to Björk; GIC: glass ionomer cement; GOPEX: glass ionomer open exposure technique; PDC(s): palatally displaced canine(s); RCT: randomized controlled trial.

Study ID	Study Design	Country	Population	Sample Size	Follow up	Primary outcomes	Closed technique				Open technique			
							Flap & Bone	Exposure	Dressing/Attachment	Traction	Flap & Bone	Exposure	Dressing/Attachment	Traction
Björksved et al. [10]	Multicentre RCT	Sweden	9-16 years old, PDCs planned for surgical exposure, max DS2M1–DS3M2.	119 patients (59 open, 60 closed)	4 weeks	Surgery time, complications, and patients’ perceptions	Full-thickness mucoperiosteal flap raised, bone covering canine crown removed.	No tissue window removed.	An attachment with a chain was bonded to the exposed canine.	Canine left to erupt spontaneously without orthodontic force.	Full-thickness mucoperiosteal flap raised, bone covering canine crown removed.	A window of mucoperiosteal tissue overlying the tooth was removed.	Light-cured GIC applied to the canine crown extending 2 mm above mucosa.	Orthodontic traction was initiated within 2 weeks post-surgery.
Björksved et al. [24]	Multicentre RCT	Sweden	9-16 years old, PDCs planned for surgical exposure, max DS2M1–DS3M2.	119 patients (59 open, 60 closed)	26 and 52 weeks	Periodontal status, eruption time, treatment duration, and cost-effectiveness	Full-thickness mucoperiosteal flap raised, bone covering canine crown removed.	No tissue window removed.	An attachment with a chain was bonded to the exposed canine.	Canine left to erupt spontaneously without orthodontic force.	Full-thickness mucoperiosteal flap raised, bone covering canine crown removed.	A window of mucoperiosteal tissue overlying the tooth was removed.	Light-cured GIC applied to the canine crown extending 2 mm above mucosa.	Orthodontic traction was initiated within 2 weeks post-surgery.
Dahlén et al. [11]	Single-centre RCT	Sweden	Patients ≤18 years old with a unilateral PDC in sectors 2–5.	92 patients (43 open, 40 closed)	4 weeks	Pain, complications, surgery time	Mucoperiosteal flap raised, bone removed using a noncrosscut fissure bur.	A 2 mm biopsy punch was used to perforate the flap.	An eyelet was bonded with light-cured flowable composite. Flap repositioned.	Orthodontic traction was applied subsequently.	Mucoperiosteal flap raised, bone removed using a noncrosscut fissure bur.	A 6 mm biopsy punch was used to create an opening through the flap.	Flap sutured back into original position. Glass ionomer cement (GIC) applied.	Tooth erupts spontaneously.
Færøvig et al. [12]	Two-centre RCT	Norway and Sweden	Patients ≤16 years old with unilateral or bilateral PDCs, canine cusp tip in sectors 2–5.	100 patients (45 open, 47 closed)	3 weeks	Pain, analgesic intake, complications, surgery time	Bone removed to the widest curvature of the crown. Flap fully repositioned.	No tissue window removed.	Attachment with chain bonded to the crown. Flap fully repositioned and sutured.	Forced orthodontic movement initiated 2–3 weeks post-surgery.	Mucoperiosteal flap elevated, bone removed to the widest curvature of the crown.	Differed by center (excised or pushed back).	Coe-pak surgical dressing placed over the exposed area and retained with sutures.	Canine left to erupt spontaneously above the mucosa without orthodontic assistance.
Gharaibeh et al. [25]	Single-centre RCT	Jordan	Patients with unilateral palatally impacted maxillary canines.	32 patients (16 open, 16 closed)	1 week	Pain, surgery time	Standard palatal mucoperiosteal flap raised; bone removed with rotary instruments if needed.	No tissue window removed.	A gold chain was bonded to the available surface of the crown. Flap sutured back.	Orthodontic traction started 1 week after surgery.	Standard palatal mucoperiosteal flap raised; bone removed with rotary instruments if needed.	Adequate amount of palatal flap over the crown cut with a surgical blade.	Antiseptic gauze pack sutured into the defect with 3/0 black silk suture. Pack removed after 1 week, lingual button bonded.	Orthodontic traction started 1 week after surgery.
Parkin et al. [26]	Multicentre RCT	United Kingdom	Patients ≤20 years old with unilateral PDC requiring surgical exposure, good oral hygiene.	81 patients (40 open, 41 closed)	10 days	Pain, surgery time	Palatal flap raised, limited bone removal.	No tissue window removed.	An eyelet attachment with a gold chain was bonded to the crown. Flap sutured back.	Canine aligned from below the mucosa.	Palatal flap raised, bone removed to expose the largest diameter of the ectopic canine crown.	Palatal mucosa surgically excised, standardized using a preformed wire template.	Surgical gauze soaked in Whitehead varnish or Coe-pack surgical dressing was sutured in place. Pack removed 10 days later.	Canine subsequently orthodontically aligned above the mucosa.
Parkin et al. [28]	Multicentre RCT	United Kingdom	Patients ≤20 years old with unilateral PDC requiring surgical exposure, good oral hygiene.	62 patients	3 & 12 months	Clinical attachment level, periodontal status	Palatal flap raised, limited bone removal.	No tissue window removed.	An eyelet attachment with a gold chain was bonded to the crown. Flap sutured back.	Canine aligned from below the mucosa.	Palatal flap raised, bone removed to expose the largest diameter of the ectopic canine crown.	Palatal mucosa surgically excised, standardized using a preformed wire template.	Surgical gauze soaked in Whitehead varnish or Coe-pack surgical dressing was sutured in place. Pack removed 10 days later.	Canine subsequently orthodontically aligned above the mucosa.
Parkin et al. [27]	Multicentre RCT	United Kingdom	Patients ≤20 years old with unilateral PDC requiring surgical exposure, good oral hygiene.	67 patients	3 & 12 months	Esthetic outcomes	Palatal flap raised, limited bone removal.	No tissue window removed.	An eyelet attachment with a gold chain was bonded to the crown. Flap sutured back.	Canine aligned from below the mucosa.	Palatal flap raised, bone removed to expose the largest diameter of the ectopic canine crown.	Palatal mucosa surgically excised, standardized using a preformed wire template.	Surgical gauze soaked in Whitehead varnish or Coe-pack surgical dressing was sutured in place. Pack removed 10 days later.	Canine subsequently orthodontically aligned above the mucosa.
Smailiene et al. [29]	Prospective controlled clinical study	Lithuania	Patients with unilateral palatally impacted maxillary canines, complete root development, and good oral hygiene.	43 patients (22 open, 21 closed)	3–6 months	Periodontal status, treatment duration	Closed flap technique according to Kokich and Mathews (1993).	No tissue window removed.	Attachment bonded beneath the flap.	Extrusion of the impacted tooth was initiated 1 week after surgery using a ballista loop.	Surgical approach according to Kokich and Mathews (1993).	Tissue removed to expose the crown.	Periodontal dressing applied. Dressing removed 1 week after surgery.	Tooth allowed to erupt spontaneously.

**Table 3:** Baseline characteristics of the included populations

Study ID	Intervention	Number	Age (year), Mean (SD)	Male, N (%)	Unilateral/Bilateral, N (%)	Sector, N (%)	Angulation of canine (degree), Mean (SD)	Vertical position (mm), Mean (SD)	Previous orthodontic treatment, N (%)
Björksved et al. [10]	Open exposure	59	13.3 (1.3)	24 (40.7)	Unilateral: 44 (74.6), Bilateral: 15 (25.4)	Sector III: 36 (61.0), Sector IV: 23 (39.0)	32.4 (8.2)	9.0 (2.0)	15 (25.9)
	Closed exposure	60	13.5 (1.6)	20 (33.3)	Unilateral: 45 (75.0), Bilateral: 15 (25.0)	Sector III: 31 (51.7), Sector IV: 29 (48.3)	32.4 (7.53)	8.9 (2.3)	27 (45.8)
Björksved et al. [24]	Open exposure	59	13.3 (1.3)	24 (40.7)	Unilateral: 44 (74.6), Bilateral: 15 (25.4)	Sector III: 36 (61.0), Sector IV: 23 (39.0)	32.4 (8.2)	9.0 (2.0)	15 (25.9)
	Closed exposure	60	13.5 (1.6)	20 (33.3)	Unilateral: 45 (75.0), Bilateral: 15 (25.0)	Sector III: 31 (51.7), Sector IV: 29 (48.3)	32.4 (7.53)	8.9 (2.3)	27 (45.8)
Dahlén et al. [11]	Open exposure	43	13.8 (1.5)	16 (37.2)	Unilateral: 43 (100)	Sector II: 8 (18.6), Sector III: 12 (27.9), Sector IV: 16 (37.2), Sector V: 7 (16.3)	30.0 (10.9)	11.8 (2.2)	NA
	Closed exposure	40	13.6 (1.2)	18 (45.0)	Unilateral: 40 (100)	Sector II: 3 (7.5), Sector III: 18 (45.0), Sector IV: 10 (25.0), Sector V: 9 (22.5)	33.9 (13.5)	11.7 (2.5)	NA
Færøvig et al. [12]	Open exposure	45	12.9 (1.4)	15 (33.3)	Unilateral: 35 (77.8), Bilateral: 10 (22.2)	Sector II: 3 (6.7), Sector III: 29 (64.4), Sector IV: 13 (28.9)	30.6 (7.0)	13.8 (2.3)	0
	Closed exposure	47	13.1 (1.4)	17 (36.2)	Unilateral: 36 (76.6), Bilateral: 11 (23.4)	Sector II: 5 (10.6), Sector III: 25 (53.2), Sector IV: 17 (36.2)	31.3 (10.2)	13.6 (2.8)	0
Gharaibeh et al. [25]	Open exposure	16	17.3 (4.5)	2 (12.5)	Unilateral: 16 (100)	NA	NA	NA	NA
	Closed exposure	16	17.6 (2.4)	2 (12.5)	Unilateral: 16 (100)	NA	NA	NA	NA
Parkin et al. [26]	Open exposure	40	14.3 (1.3)	13 (32.5)	Unilateral: 40 (100)	NA	33.1 (14.4)	13.6 (3.1)	NA
	Closed exposure	41	14.1 (1.6)	16 (39.0)	Unilateral: 41 (100)	NA	31.9 (13.3)	13.2 (2.8)	NA
Parkin et al. [28]	Open exposure	33	14.3 (1.3)	11 (33.3)	Unilateral: 33 (100)	NA	33.1 (14.4)	13.6 (3.1)	NA
	Closed exposure	29	14.1 (1.6)	12 (41.4)	Unilateral: 29 (100)	NA	31.9 (13.3)	13.2 (2.8)	NA
Parkin et al. [27]	Open exposure	34	14.3 (1.3)	11 (32.4)	Unilateral: 34 (100)	NA	33.1 (14.4)	13.6 (3.1)	NA
	Closed exposure	33	14.1 (1.6)	13 (39.4)	Unilateral: 33 (100)	NA	31.9 (13.3)	13.2 (2.8)	NA
Smailiene et al. [29]	Open exposure	22	15.46 (3.28)	8 (18.6)	Unilateral: 22 (100)	H1: 7 (31.8), H2: 7 (31.8), H3: 4 (18.2), H4: 4 (18.2)	38.5 (14.02)	NA	0
	Closed exposure	21	16.15 (2.79)	8 (18.6)	Unilateral: 21 (100)	H1: 7 (33.3), H2: 9 (42.9), H3: 4 (19.0), H4: 1 (4.8)	34.33 (14.57)	NA	0

Quality Assessment

**Figure 2:**
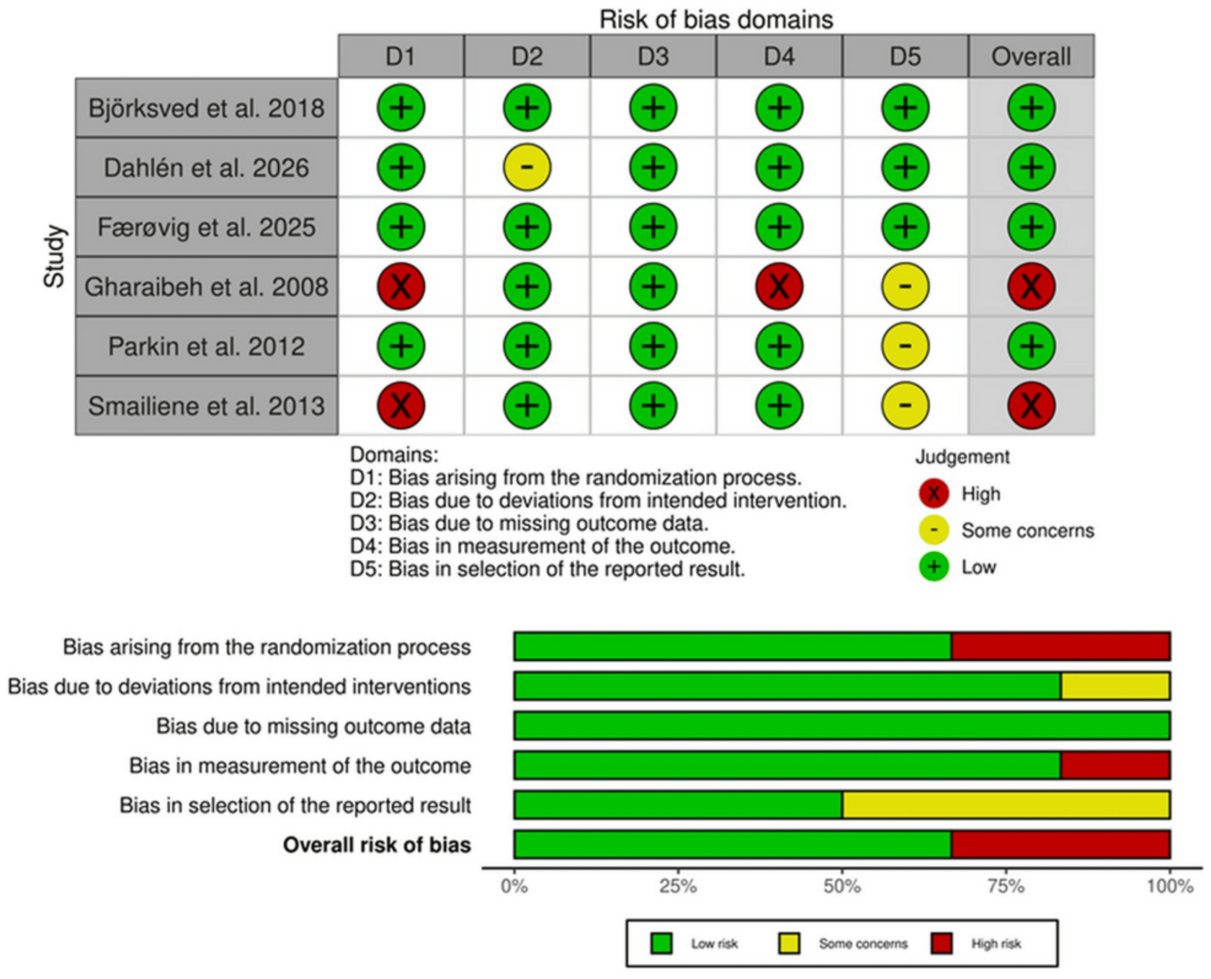
Summary and traffic light plots of the risk of bias of the included randomized controlled trials

Risk of bias assessment for the included trials using the Cochrane ROB2 tool showed variable quality across the different domains, as summarized in Table 3 (Figure 2). The trials by Björksved et al., Færøvig et al., Dahlén et al., and Parkin et al. demonstrated an overall low risk of bias [10-12, 26]. In contrast, Smailiene et al. and Gharaibeh et al. were assessed as having a high risk of bias, primarily due to concerns regarding the randomization process and allocation concealment [25, 29].

Outcomes

Primary outcomes: The primary outcomes assessed in this meta-analysis were postoperative pain and discomfort following closed versus open surgical exposure of palatally displaced canines.

**Figure 3:**
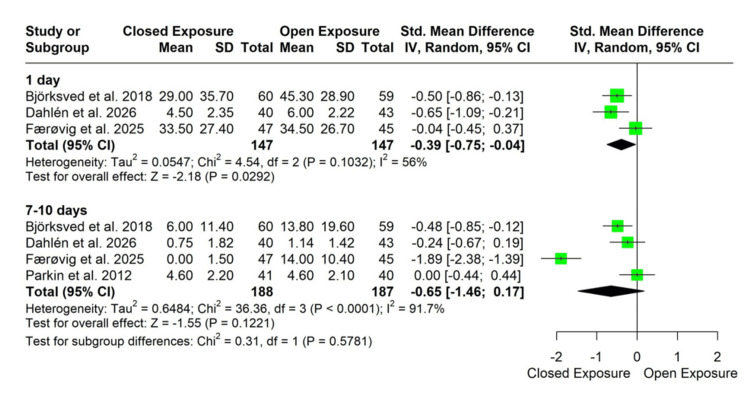
Forest plot comparing postoperative pain scores between closed and open exposure techniques at 1 day and 7–10 days

**Figure 4:**
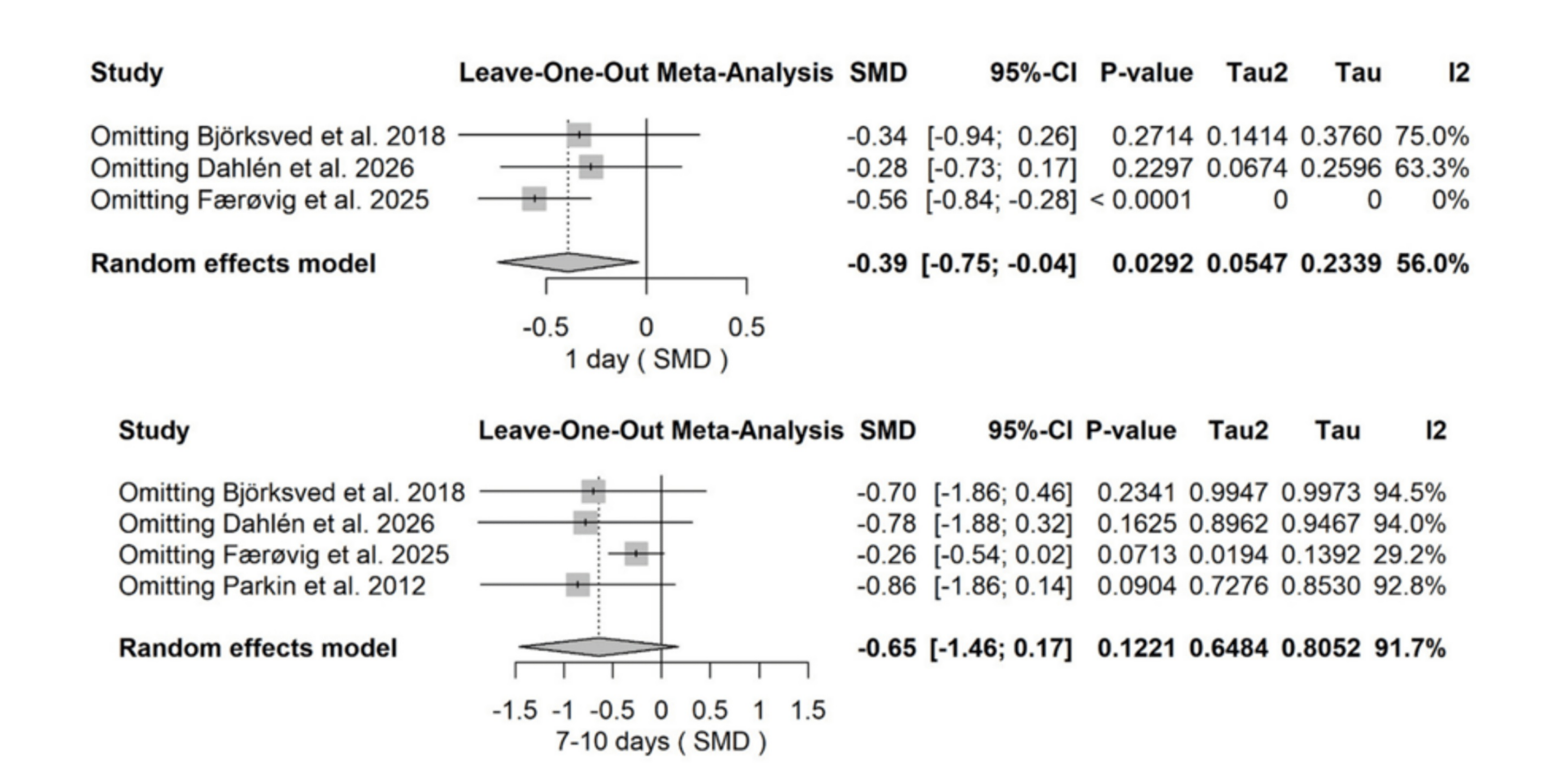
Leave-one-out sensitivity analysis for postoperative pain scores at 1 day and 7–10 days [10-12, 26]

**Figure 5:**
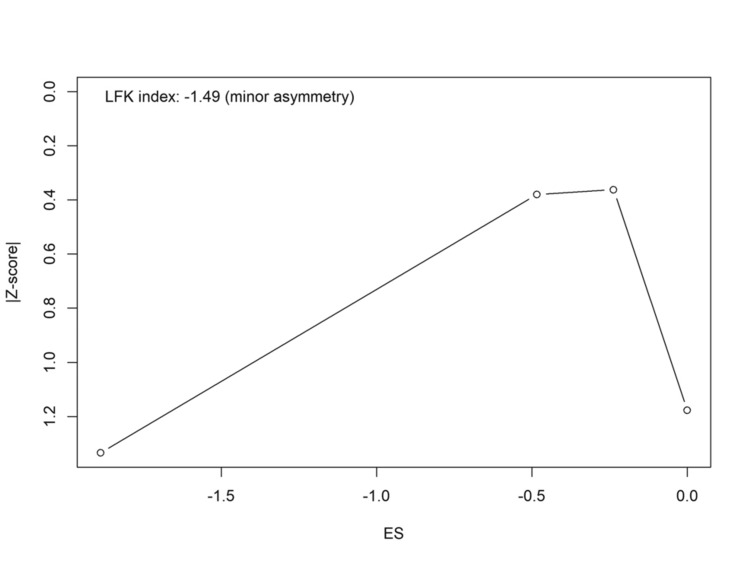
Doi plot assessing publication bias for postoperative pain at 7–10 days

**Figure 6:**
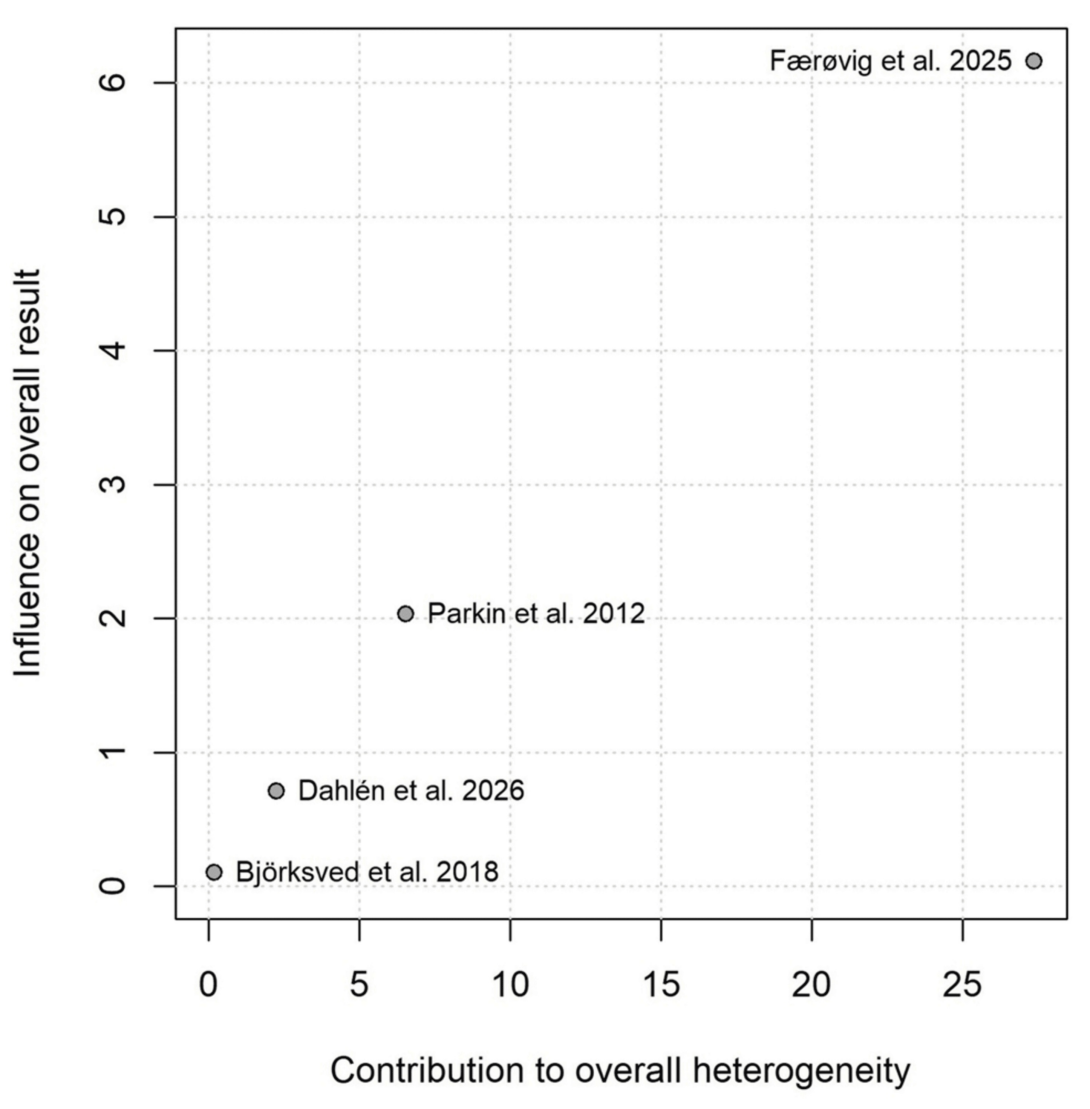
Baujat plot assessing the contribution of individual studies to between-study heterogeneity and their influence on the overall effect size for postoperative pain (7–10 days) [10-12, 26]

**Figure 7:**
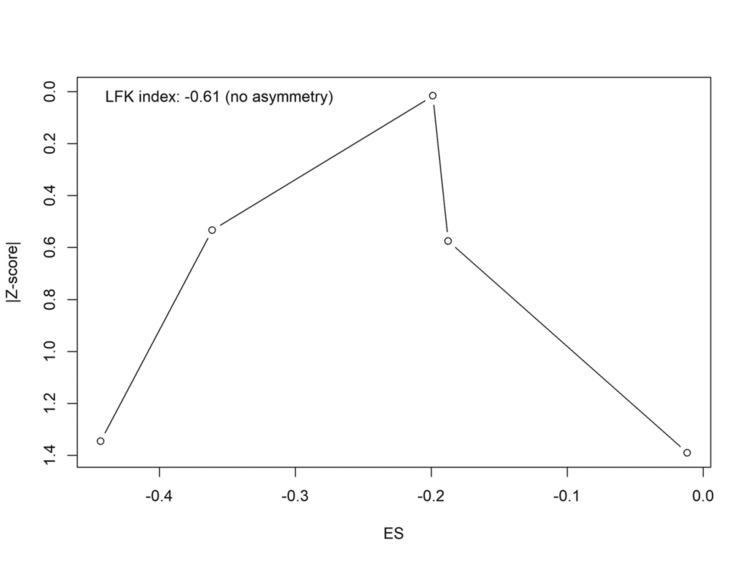
Doi plot assessing publication bias for postoperative discomfort

**Figure 8:**
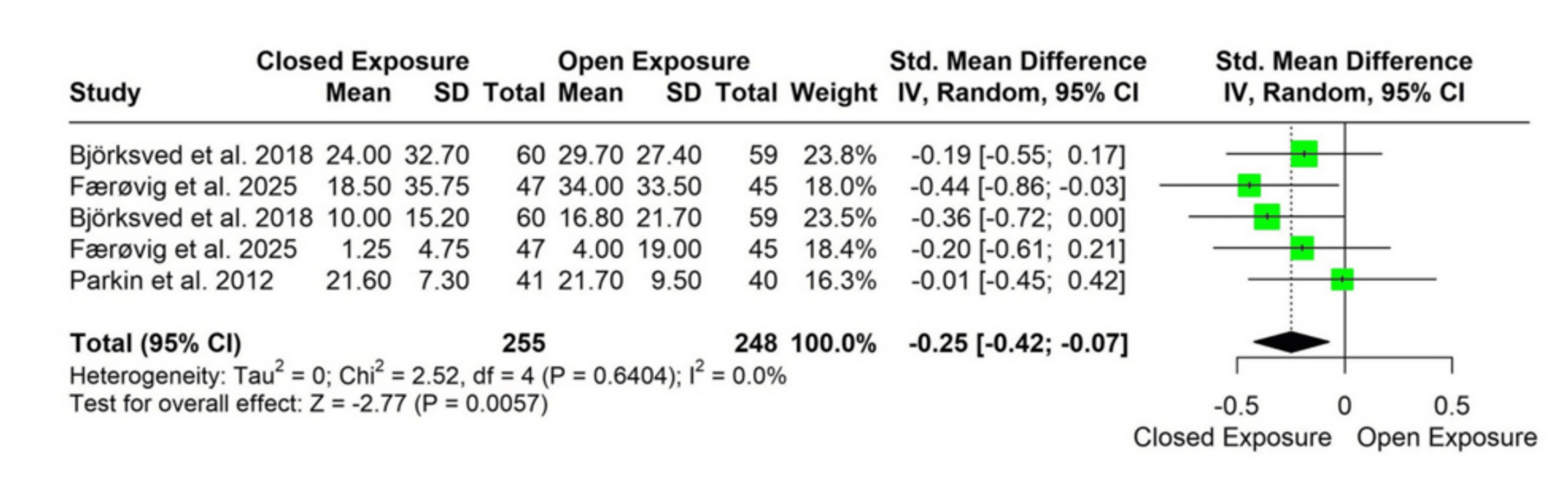
Forest plot comparing postoperative discomfort between closed and open exposure techniques [10,12,26]

a) Pain - The pooled analysis of pain scores demonstrated a time-dependent effect between closed and open exposure techniques. On day one, there was a statistically significant reduction in pain favoring the closed-exposure group (SMD=-0.39, 95% CI:-0.75 to -0.04; P=0.029), with moderate heterogeneity (I²=56%). In contrast, at seven to 10 days, although the point estimate continued to favor the closed exposure group, the difference was not statistically significant (SMD=-0.65, 95% CI: -1.46 to 0.17; P=0.122), and substantial heterogeneity was observed (I²=91.7%). Importantly, there was no significant difference between subgroups based on follow-up duration (χ²=0.31, P=0.578), suggesting that the effect of exposure type on pain reduction did not significantly vary over time (Figure 3). The leave-one-out sensitivity analysis showed that excluding Færøvig et al. [12] resulted in complete elimination of heterogeneity for the one-day outcome (I²=0%), indicating that this study was the sole contributor to between-study variability. Importantly, the pooled effect size demonstrated a statistically significant benefit in favor of the closed exposure technique (SMD=-0.56, 95% CI:-0.84 to -0.28; P<0.0001). For the seven- to 10-day outcome, exclusion of Færøvig et al. [12] markedly reduced heterogeneity (I² decreased from 91.7% to 29.2%) and shifted the effect toward significance (SMD=-0.26, 95% CI: -0.54 to 0.02; P=0.071), indicating that this study was a major source of heterogeneity (Figure 4). For the seven-to 10-day outcome, the Doi plot for the pain outcome demonstrates minor asymmetry, as indicated by an LFK index of -1.49 (Figure 5). For the seven-to 10-day outcome, the Baujat plot indicates that Færøvig et al. [12] is the primary contributor to between-study heterogeneity, with the highest contribution and influence on the overall pooled estimate (Figure 6).

b) Discomfort - The pooled analysis of discomfort demonstrated a statistically significant reduction favoring the closed exposure technique (SMD=-0.25, 95% CI:-0.42 to -0.07; P=0.0057). Importantly, there was no observed heterogeneity among the included studies (I²=0%, τ²=0), indicating a high level of consistency in the effect estimates across studies (Figure 7). The Doi plot for discomfort demonstrates no evidence of asymmetry, as indicated by an LFK index of -0.61 (Figure 8).

Secondary outcomes: The secondary outcomes evaluated included surgery time, analgesic consumption, gingival recession, eruption time, and overall complications.

**Figure 9:**
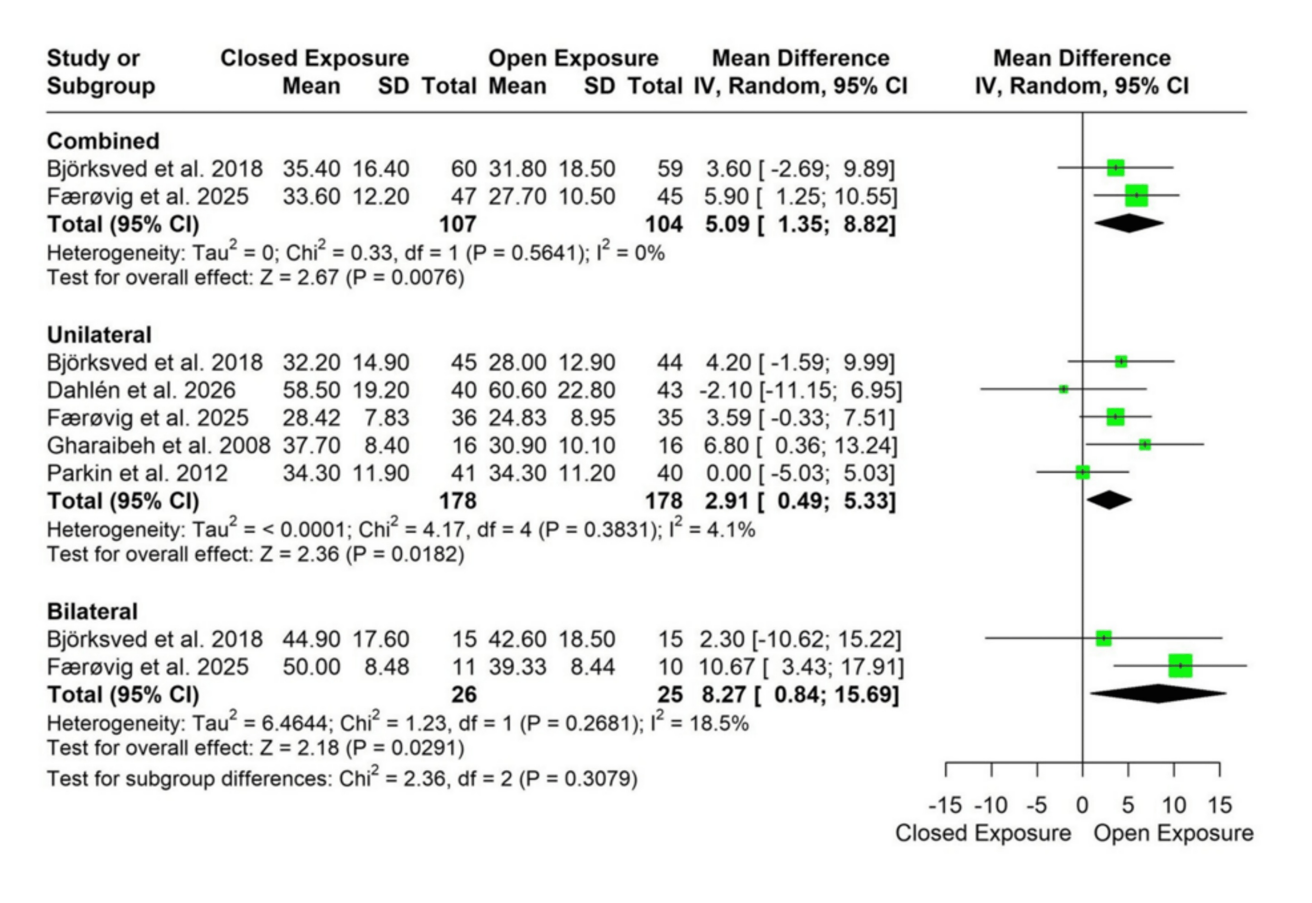
Forest plot comparing surgical time (minutes) between closed and open exposure techniques, including subgroup analyses for combined, unilateral, and bilateral cases [10-12, 25, 26]

**Figure 10:**
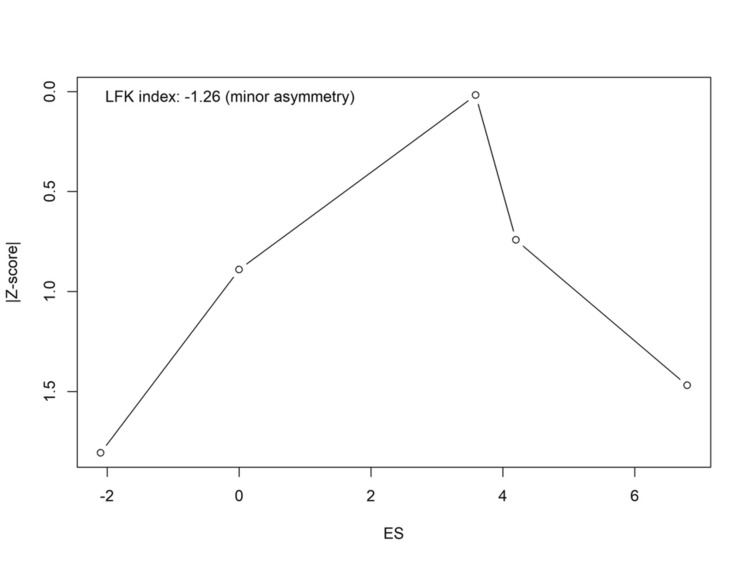
Doi plot assessing publication bias for surgical time (unilateral cases)

**Figure 11:**
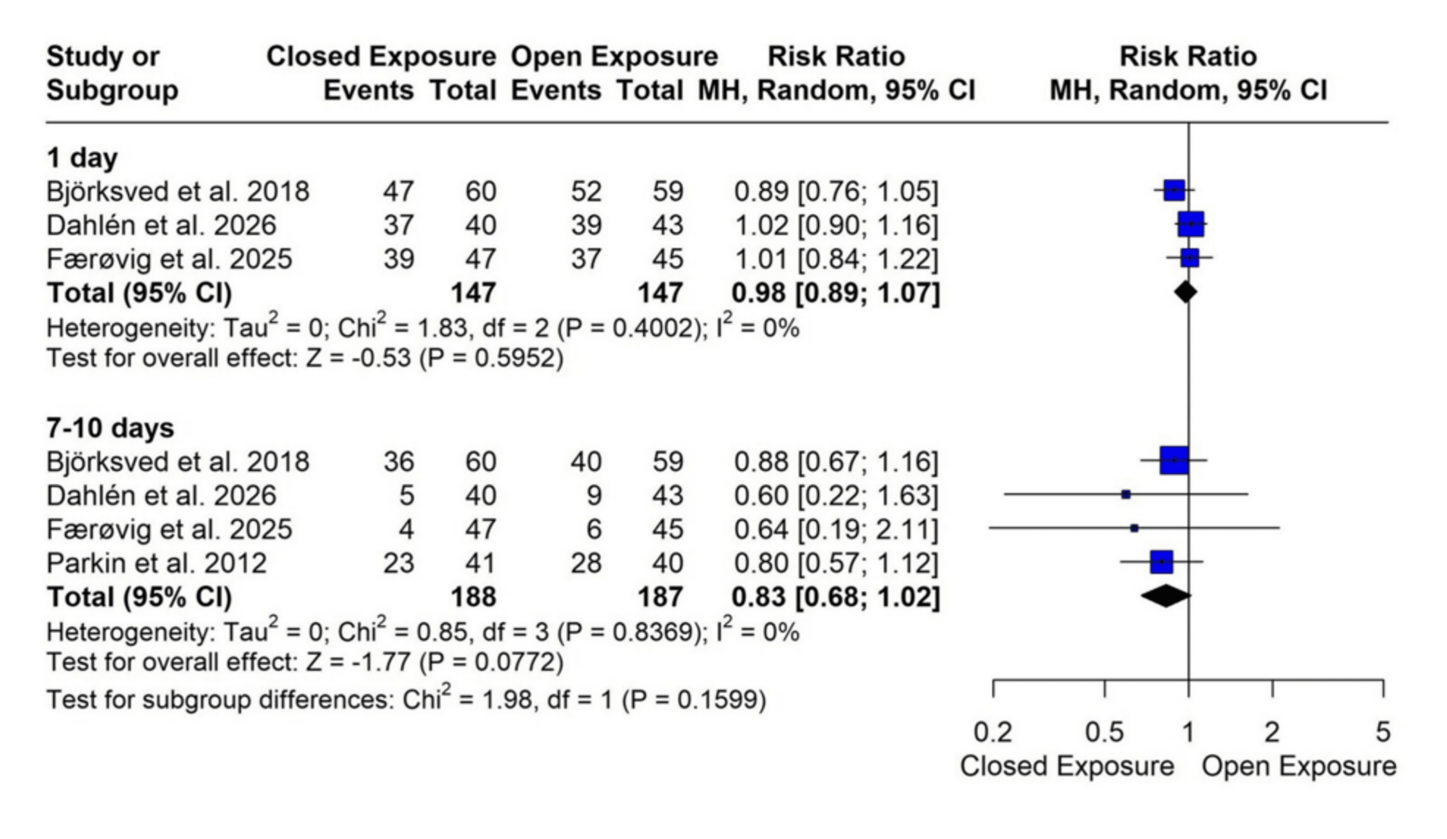
Forest plot comparing analgesic consumption between closed and open exposure techniques at 1 day and 7–10 days [10-12, 26]

**Figure 12:**
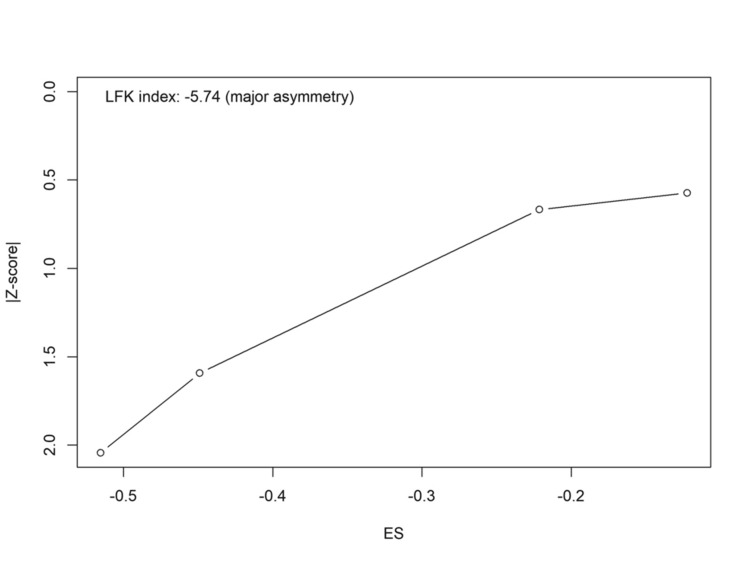
Doi plot assessing publication bias for analgesic consumption at 7–10 days

**Figure 13:**
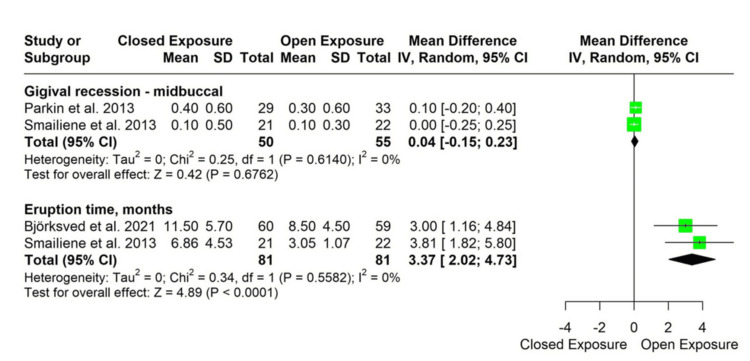
Forest plot comparing midbuccal gingival recession and eruption time (months) between closed and open exposure techniques [10, 26, 29]

**Figure 14:**
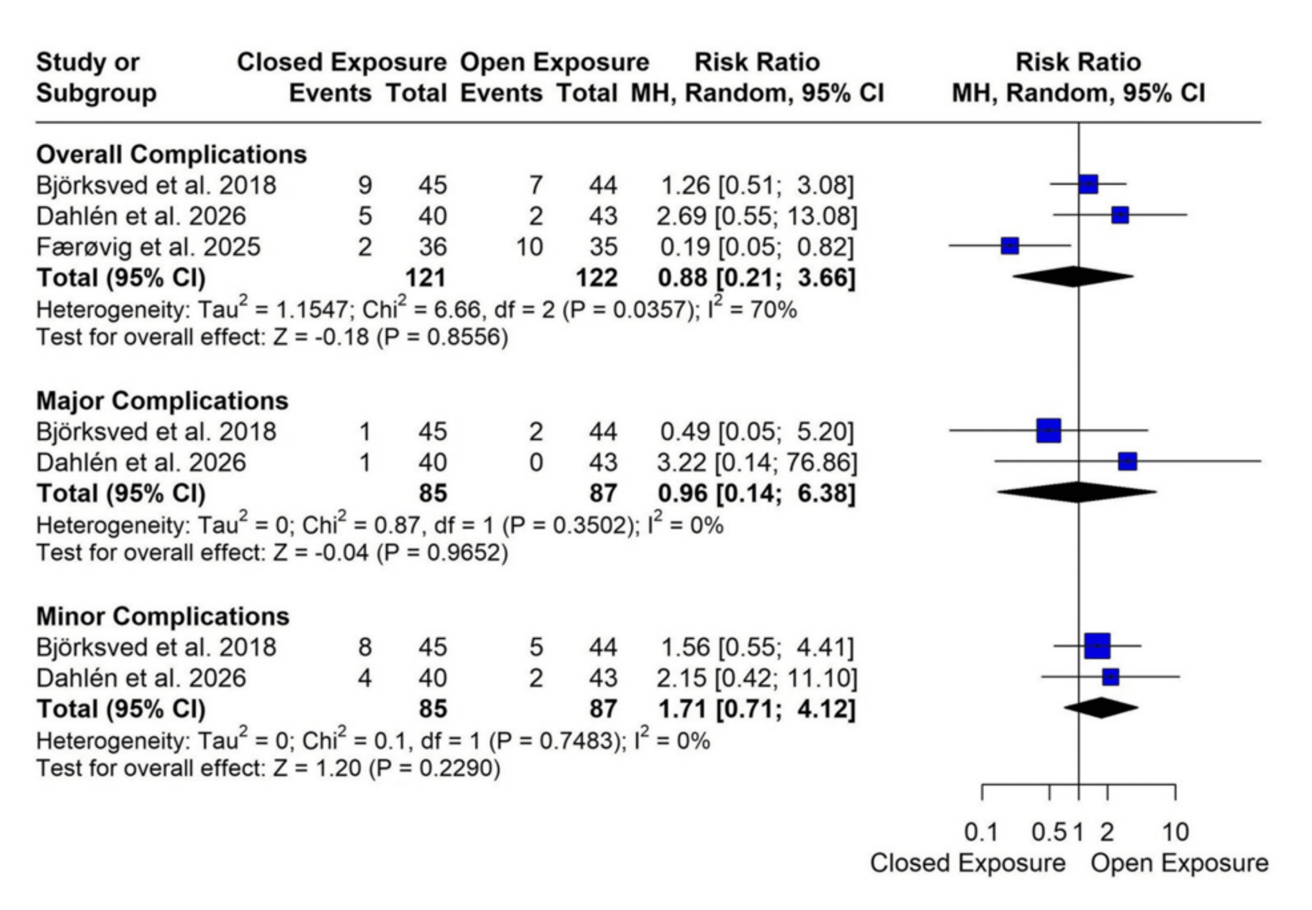
Forest plot comparing overall, major, and minor complications between closed and open exposure techniques [10-12]

**Figure 15:**
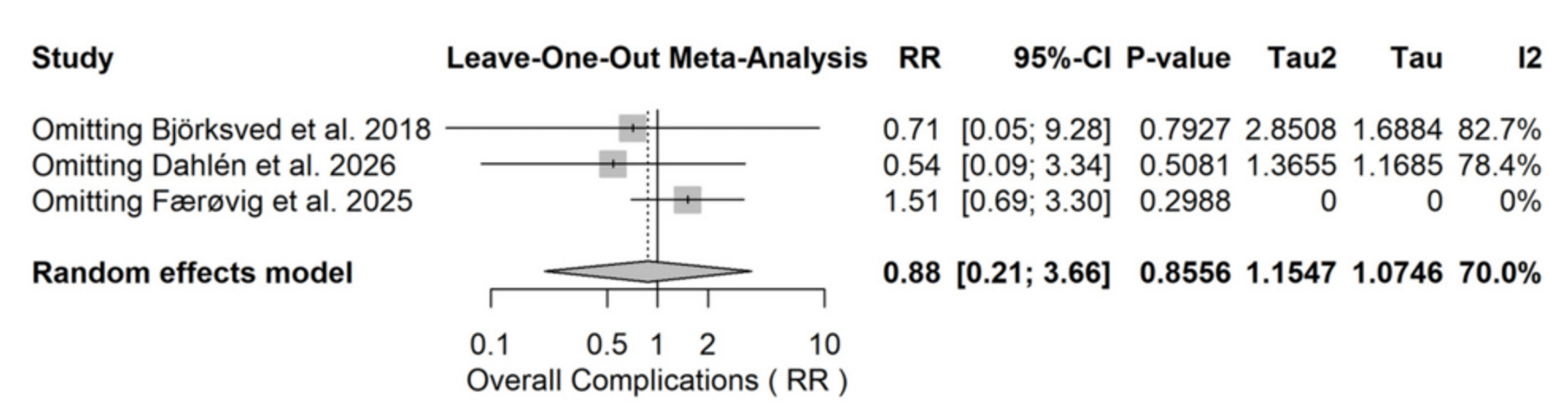
Leave-one-out sensitivity analysis for overall complications [10-12]

a) Surgery Time (min) - The pooled analysis of surgical time demonstrated a significantly longer duration associated with the closed exposure technique compared to open exposure across all subgroups. In the combined analysis, closed exposure was associated with an increase of 5.09 minutes (MD=5.09, 95% CI: 1.35 to 8.82; P=0.0076), with no observed heterogeneity (I²=0%), indicating highly consistent findings across studies. Subgroup analyses showed similar results. In unilateral cases, closed exposure resulted in a significantly longer surgical time (MD=2.91, 95% CI: 0.49 to 5.33; P=0.018), with minimal heterogeneity (I²=4.1%). In bilateral cases, the effect was more pronounced, with an increase of 8.27 minutes (MD=8.27, 95% CI: 0.84 to 15.69; P=0.029), although with slightly higher but still low heterogeneity (I²=18.5%). Importantly, there was no statistically significant difference between subgroups (P=0.308), suggesting that the increase in operative time with closed exposure is consistent regardless of laterality (Figure 9). The Doi plot for surgical time (unilateral cases) demonstrates minor asymmetry, as indicated by an LFK index of -1.26 (Figure 10).

b) Analgesic Consumption - At day one, the overall effect estimate was not statistically significant (RR=0.98, 95% CI:0.89 to 1.07; P=0.595), with no observed heterogeneity (I²=0%), indicating consistent findings across studies. Similarly, at seven to 10 days, the pooled estimate suggested a trend toward reduced analgesic use with the closed exposure technique (RR=0.83, 95% CI: 0.68 to 1.02), but this did not reach statistical significance (P=0.077). Heterogeneity remained absent (I²=0%), further supporting the consistency of the results. Additionally, there was no significant difference between subgroups based on follow-up duration (P=0.160), indicating that the effect of exposure technique on analgesic consumption did not vary over time (Figure 11). For the seven- to 10-day period, the Doi plot for analgesic consumption demonstrates major asymmetry, as indicated by an LFK index of -5.74 (Figure 12).

c) Gingival Recession (midbuccal) and Eruption Time (months) - The analysis of gingival recession (midbuccal) demonstrated no significant difference between closed and open exposure techniques (MD=0.04, 95% CI:−0.15 to 0.23; P=0.676), with no observed heterogeneity (I²=0%) (Figure 13). In contrast, the pooled analysis of eruption time showed that the closed exposure technique was associated with a significantly longer eruption duration compared to open exposure (MD=3.37 months, 95% CI:2.02 to 4.73; P<0.0001). No heterogeneity was observed (I²=0%) (Figure 13).

d) Overall Complication - The pooled analysis of overall complications showed no significant difference between closed and open exposure techniques (RR=0.88, 95% CI:0.21 to 3.66; P=0.856). However, there was substantial heterogeneity among the included studies (I²=70%), indicating considerable variability in effect estimates and reducing confidence in the pooled result (Figure 14). The leave-one-out sensitivity analysis showed that excluding Færøvig et al. [12] resulted in complete elimination of heterogeneity (I²=0%), indicating that this study was the main source of between-study variability in the overall complications analysis. Under this condition of no heterogeneity, the pooled effect estimate shifted to RR=1.51 (95% CI:0.69 to 3.30) (Figure 15). Subgroup analyses provided further insight. For major complications, no significant difference was observed between groups (RR=0.96, 95% CI:0.14 to 6.38; P=0.965), with no heterogeneity (I²=0%), suggesting consistent findings across studies. Similarly, for minor complications, the pooled estimate did not reach statistical significance (RR=1.71, 95% CI:0.71 to 4.12; P=0.229), again with no observed heterogeneity (I²=0%) (Figure 14).

Discussion

Summary of Findings

This systematic review and meta-analysis compared open versus closed exposure techniques for palatally displaced canines, including data from six studies with 450 participants. The results indicate that closed surgical exposure provides significant advantages regarding early postoperative pain (at day one) and overall discomfort, with consistent effect estimates across studies. However, these benefits are accompanied by significantly longer surgical time, with approximately five minutes longer overall, and eight minutes longer for bilateral cases, as well as substantially prolonged eruption time. No significant differences were observed between the two techniques for analgesic consumption, gingival recession, or total complications, although considerable heterogeneity for overall complications was largely driven by a single study.

Interpretation of Findings

The finding that closed exposure is associated with less early postoperative pain is clinically meaningful. One possible explanation for the increased pain with open exposure is that this technique results in a larger wound surface associated with the GIC compared to closed exposure, where the opening need not be larger than what is required for a chain to pass through [11]. Dental fear, which is linked to higher self-reported pain [29], was more frequent in the GOPEX group (four vs. one patient above the Children’s Fear Survey Schedule - Dental Subscale (CFSS-DC) clinical threshold) [30]. Although this may have influenced postoperative pain, the difference was not statistically significant (P=0.07) and is unlikely to have had a meaningful impact [11].

The open surgical technique does not require an orthodontist to bond a chain to the impacted canine's crown, so it is expected to require less operative time than the closed approach. In the present study, the mean surgical duration for the open technique was significantly shorter than for the closed technique. Gharaibeh et al. [25] and Færøvig et al. [12] also found that surgery time was significantly shorter in the open exposure group. However, the opposite of the aforementioned finding was reported by Chaushu et al. [31], who observed significantly shorter surgical duration in the closed group. Inconsistent conclusions are further illustrated by Parkin et al. [26] and Björksved et al. [10], who found no difference between the two techniques; the former used a surgical dressing, while the latter used GIC.

Previous studies have reported greater pain following open surgical exposure [10, 12, 31], whereas others found no differences in pain scores between open and closed exposure [24, 25]. Pain intensity in these studies was assessed at a single time point [25], on postoperative days one and seven [10], or daily for seven days after surgery [10, 24, 31].

An interesting finding was that the mean time for impacted canine eruption in the open technique group was significantly shorter than in the closed technique group. A previous RCT hypothesized that this shorter duration may be partly explained by the fact that the teeth in open exposure had a more superficial position compared to those in closed exposure (81.8% of teeth in group 1 were located in sector V1 versus 61.9% in group 2), although this difference did not reach statistical significance [28]. Nevertheless, total treatment duration was similar between the two groups. This may be explained by differences in the treatment process: palatally impacted canines allowed to erupt naturally may emerge more quickly since they do not have to pass through soft tissue; however, such teeth typically erupt in a more anterior and palatal position. Consequently, subsequent alignment may require additional time [28].

In our analysis, closed exposure required approximately 3.4 months longer for the canine to reach the occlusal plane or become visible. The intra-alveolar position of a PDC has been suggested to influence treatment duration. In a retrospective study of 45 patients with PDCs treated with closed exposure, the authors found a significant relationship between treatment time and the horizontal position of the canine crown relative to adjacent teeth and the maxillary dental midline. In contrast, treatment duration was not associated with the angulation or vertical height of the PDC [32]. That study used the anatomic landmark "canine tip" to define the horizontal position of the PDC according to sectors on panoramic radiographs. However, baseline data from another trial on horizontal position (sector) showed no effect on total treatment time [23].

Comparing treatment duration outcomes across studies in relation to PDC position on panoramic radiographs is challenging due to the use of different, or sometimes absent, defined anatomical landmarks. Studies that classify PDCs using various criteria, such as "canine crown" [33], "any overlap of the PDC with adjacent teeth" [25], or "cusp tip" [34], may yield differing results because the initial position, therefore the severity of displacement, may vary. The criterion "any overlap" may allow inclusion of PDCs in sector II, which have more favorable eruption paths, whereas the "cusp tip" definition typically includes PDCs in sectors III and higher, representing more severe displacement.

Regarding complications, the lack of significant difference between techniques is reassuring for clinicians and patients. Both major and minor complications were rare across studies, with no technique demonstrating superiority. The absence of significant difference in gingival recession (midbuccal) is an important periodontal outcome. Our pooled analysis, with no heterogeneity, provides preliminary evidence that this is not the case. Both techniques appear to result in similar periodontal outcomes when performed by experienced clinicians.

Our findings update and extend the previous Cochrane review, which included only three RCTs and was unable to perform meta-analyses for several outcomes due to insufficient data. The Cochrane review concluded that there was insufficient evidence to determine whether open or closed exposure was superior [2]. Our findings are in line with the results of the individual studies included in this review. Dahlén et al. observed no significant differences in overall complication rates. However, they reported greater pain in the open exposure group during the first week after surgery. Notably, the authors reported that pain scores and analgesic use did not differ between the two groups over the entire postoperative period [11]. Færøvig et al. found that open surgical exposure required less operative time [12].

Strengths and Limitations

To our knowledge, this is the most comprehensive meta-analysis to compare open and closed surgical exposure techniques for PDCs, incorporating data from most recent RCTs. We conducted a recent thorough search across four major databases. Rigorous statistical methods were applied. Sensitivity analyses and Baujat plots were employed to identify sources of heterogeneity. Also, publication bias was assessed using DOI plots and the LFK index given the limited number of studies.

However, several limitations must be acknowledged. First, the number of included studies was small, and the total sample size is modest for a meta-analysis. Second, substantial heterogeneity was present for some outcomes, although sensitivity analyses identified the source of this heterogeneity. Third, outcome definitions varied across studies, necessitating the use of SMD for some analyses. Fourth, the risk of bias in two included studies was high. Fifth, long-term outcomes were not consistently reported across studies and could not be meta-analyzed. Finally, variation in open technique protocols may have impacted total treatment time and eruption time outcomes.

Clinical Implications and Future Recommendations

Closed exposure appears preferable when minimizing early postoperative pain and discomfort is a priority for the patient. However, this benefit is combined with longer surgical and eruption times. These benefits and drawbacks should be explained to patients and their families and may be integrated as part of informed consent. Our findings of comparable complication rates and periodontal outcomes provide reassurance that neither technique is associated with greater harm. Future large-scale, multicenter RCTs with longer follow-up are needed to validate our findings.

## Conclusions

Closed surgical exposure for palatally displaced canines might offer advantages in reducing early postoperative pain and discomfort compared to open exposure, but at the cost of longer surgical time and substantially longer eruption time. Both techniques appear comparable regarding analgesic consumption, gingival recession, and complications. Future high-quality RCTs with standardized outcome definitions and longer follow-up periods are needed to validate these findings.
